# Impact of Incorporating Dried Chaga Mushroom (*Inonotus obliquus*) into Gluten-Free Bread on Its Antioxidant and Sensory Characteristics

**DOI:** 10.3390/molecules29163801

**Published:** 2024-08-10

**Authors:** Zbigniew Kobus, Monika Krzywicka, Agata Blicharz-Kania, Alicja Bosacka, Anna Pecyna, Eva Ivanišová, Katarzyna Kozłowicz, Eva Kovačiková

**Affiliations:** 1 Department of Technology Fundamentals, University of Life Sciences in Lublin, Głeboka 28, 20-612 Lublin, Poland; zbigniew.kobus@up.lublin.pl (Z.K.); alicja.bosacka@up.lublin.pl (A.B.); anna.pecyna@up.lublin.pl (A.P.); 2 Department of Biological Bases of Food and Feed Technologies, University of Life Sciences in Lublin, Głeboka 28, 20-612 Lublin, Poland; agata.kania@up.lublin.pl (A.B.-K.); katarzyna.kozlowicz@up.lublin.pl (K.K.); 3 Institute of Food Sciences, Faculty of Biotechnology and Food Sciences, Slovak University of Agriculture, Trieda Andreja Hlinku 2, 949 76 Nitra, Slovakia; eva.ivanisova@uniag.sk; 4 Food Incubator, AgroBioTech Research Centre, Slovak University of Agriculture, Trieda Andreja Hlinku 2, 949 76 Nitra, Slovakia; 5 AgroBioTech Research Centre, Slovak University of Agriculture, Trieda Andreja Hlinku 2, 949 76 Nitra, Slovakia; eva.kovacikova@uniag.sk

**Keywords:** gluten-free bread, antioxidant activity, polyphenols, bioactive compounds, chaga, sensory analysis

## Abstract

Gluten-free bread is increasingly popular among individuals with celiac disease, and The incorporation of mushroom flour offers a novel method to enhance its nutritional profile, antioxidant content, and sensory properties. This study aimed to evaluate the antioxidant and sensory characteristics of gluten-free bread with varying amounts of chaga mushroom flour (5%, 10%, 15%, 20%). The total contents of polyphenols and flavonoids were measured using a spectrophotometric method. Antioxidant activity was assessed through DPPH and FRAP methods, while textural properties were evaluated using the TPA test. Bread colour was analysed using the CIELab system, and sensory evaluation was performed by a panel of trained consumers. The results showed that gluten-free bread enriched with chaga flour had increased polyphenol and flavonoid content and enhanced antioxidant activity. The highest levels of polyphenols, flavonoids, DPPH, and FRAP activity were found in bread with 20% chaga. The addition of chaga mushroom significantly affected the bread’s hardness, cohesiveness, and chewiness. Specifically, 20% chaga flour had the most pronounced effect on hardness and elasticity, while 15% chaga flour had the greatest impact on chewiness and cohesiveness. The bread’s colour darkened with higher chaga concentrations. The results of sensory evaluation showed a negative correlation between consumer preferences and bread fortified with chaga mushroom flour. The overall consumer acceptability score indicates that only a small addition of mushroom flour (up to 10%) can be used to bake gluten-free bread.

## 1. Introduction

Bread is a basic food product that constitutes an important element of the diet of many societies around the world. It is a source of many nutrients, such as carbohydrates (mainly starch), proteins (mainly gluten), fats and some important microelements like iron, zinc, magnesium, selenium, potassium, phosphorus and manganese [1]. Nowadays, there is a growing interest in gluten-free products due to the increase in the number of cases of celiac disease. However, many commercially available gluten-free breads are of lower quality compared to their gluten-containing counterparts [2,3]. The simplest and most effective method of preventing nutrient deficiencies in gluten-free bread is to enrich it with dietary fibres, proteins, and minerals [4,5]. Currently, enriching gluten-free bread with mushrooms has become popular. Mushrooms are a source of nutrients such as polysaccharides, proteins, fats, dietary fibre, and minerals [6]. Due to their high water content (approximately 80–90%), the fruiting bodies of medicinal mushrooms have a low caloric value (50–70 kcal/100 g) [7,8]. Incorporating mushroom raw materials into gluten-free bread is crucial for enhancing its nutritional value, antioxidant properties, and sensory characteristics.

Several reports have explored the addition of mushroom flour to gluten-free bread. Mushroom flour can significantly increase its nutritional value and antioxidant activity, making it a healthier option for consumers compared to standard products. Gluten-free bread with the addition of mushroom powder presents an interesting dietary option for people on a gluten-free diet. Additionally, mushrooms contribute other health benefits, such as increased dietary fibre, which can aid in digestive health. The literature also discusses the impact of mushroom flour on the sensory properties and overall quality of the bread. The addition of mushroom flour significantly affects sensory attributes, including aroma, taste, colour, palatability, consistency, and overall acceptability. Scientific reports have confirmed that different proportions of mushroom powder can modify the texture and rheological properties of the bread, altering the final structure of the bread crumb. The parameters assessed include hardness, elasticity, cohesiveness, chewiness, viscosity, rheological modulus, and the tangent of the bread. Additionally, some reports consider the internal porous structure of bread, which changes with increasing amounts of mushroom powder [2,9,10,11,12,13,14,15,16].

One of the raw materials that is rich in nutrients and antioxidants is chaga. It is a type of fungus that parasitizes mainly on birch trunks in the cold region of the northern hemisphere [17,18,19]. It has been used for centuries in folk medicine, particularly in Northern Europe and Russia, to treat cardiovascular diseases, diabetes, and stomach ailments [20,21]. Chaga contains approximately 2.4% protein, 10.3% polysaccharides, 4.2% reducing sugars, 1.7% fat, 67.5% dietary fibre, 10.4% ash, and 3.5% moisture in its fresh form [6].

Chaga is rich in antioxidants such as phenolic compounds [22] and melanin [23,24], which help protect cells from oxidative stress and free radical damage. Chaga has anti-inflammatory properties that may be beneficial in treating inflammation [25]. The phyto-chemical analysis of *Inonotus obliquus* also showed the presence of polysaccharides, such as beta-glucans, which have anticancer, anti-inflammatory, antiviral, antioxidant, immunomodulatory, hypoglycaemic, hypolipidemic, and hepatoprotective properties [26,27,28,29,30]. Chaga may help lower blood sugar levels and improve insulin sensitivity, which is beneficial for people with diabetes. Additionally, consuming chaga may reduce LDL cholesterol levels, thereby lowering the risk of heart disease, and support the immune system by stimulating the production of white blood cells.

Chaga and its extracts contain numerous triterpenoids that have anticancer, anti-inflammatory, antiviral, and antioxidant properties [31,32,33,34]. Chaga is rich in various macro-minerals and microelements that are essential for health, including manganese, potassium, phosphorus sodium, chlorine, sulphur, calcium, nitrogen, zinc, iron copper and selenium, vitamin B1, B2, B3, vitamin D2 vitamins A, and vitamin K [35].

Adding chaga (*Inonotus obliquus*) to bread can be an interesting way to enrich baked goods with additional health-promoting ingredients that have antioxidant, anti-inflammatory and immune system-supporting properties.

There is no information in the literature on the possibility of using chaga (*Inonotus obliquus*) preparations for food enrichment [8]. Therefore, the purpose of this study was to investigate the effect of adding chaga mushroom flour (ranging from 5 to 20%) on the antioxidant, textural, and sensory properties and to compare its properties with those of gluten-free bread of bread without additives.

## 2. Results and Discussion

### 2.1. Chemical Analysis

The results of chemical analysis in terms of bioactive substances and their antioxidant activity are presented in Table 1.

The chemical analysis indicated that the extracts from loaves of bread with the addition of chaga are characterized by a higher total polyphenol content (TPC), higher total flavonoid content (TFC), and higher antioxidant activity (DPPH and FRAP) compared to values from control bread (Table 1). The TPC for all tested samples ranges from 0.861 mg GAE/g d. m. (control bread) to 1.532 mg GAE/g d. m. for the samples with a 20% addition of chaga. The TFC ranges from 0.070 to 0.127 mg QE/g d. m. The DPPH and FRAP antioxidant activities of bread extracts range from 0.670 to 2.263 and from 1.124 to 3.360 µg TE/g d. m., respectively. The TPC, TFC, DPPH, and FRAP in dried chaga were 2.911 mg GAE/g d. m., 0.240 mg QE/g d. m., 27.530 μg TE/g d. m., and 34.329 μg TE/g d. m., respectively.

The TPC and TFC for the 20% supplemented breads were 78% and 81% higher than for the control bread, respectively. The DPPH and FRAP values for bread containing 20% addition of chaga were 238% and 199% higher than for the control bread, respectively.

Based on the ANOVA analysis and Tukey’s test, no statistically significant differences for TPC values were found between the control bread and bread containing 5% addition of chaga (*p* > 0.05). Statistically significant differences in the average TPC value were found in the case of loaves of bread with 10%, 15%, and 20% addition of chaga. However, for the TFC, DPPH, and FRAP determinations, statistically significant differences were found between all tested loaves of bread (*p* > 0.05).

In the available literature, no studies have analysed the impact of adding chaga on the total phenolic content (TPC) and total flavonoid content (TFC) values. Therefore, in the discussion, references are made to studies that examined the addition of other mushrooms to bread. Sulieman et al. [36] examined gluten-free bread enriched with mushroom flour and inulin (mushroom content of 0, 3, 6, and 9%) and showed that the total phenol content in bread with the addition of mushrooms ranges from 8.83 to 19.05 mg GAE/g d. m., while bread without mushrooms has a value of 7.79 mg GAE/g d. m. Zhang et al. [37] added different proportions of mushroom powder to wheat flour bread at 0, 2, 4, 6, and 8%, with the TPC values ranging from 28.34 (control bread) to 64.16 mg GAE/100 g (bread with 8%). Vlaic et al. [1] added boletus powder to wheat bread in proportions of 0, 3, 6, and 9% and showed that the TPC values increased from 39.88 (control bread) to 64.46 mg GAE/100 g (bread with the supplementation of 9%). Saccotelli et al. [2] introduced the addition of 15% mushroom flour (no information on the species) to gluten-free bread, resulting in a TPC value of 2.40 mg GAE/g d. m. and a TFC value of 0.45 mg QE/g d. m. Lu et al. [38] used dried shiitake mushrooms, sliced boletus mushrooms, and fresh mushrooms with 5, 10, and 15% content as an addition to white flour bread, showing a significant increase in the total phenolic content of fortified breads from 4 to 11 mg GAE/g d. m. Sławińska et al. [39] prepared the bread supplemented with *Agaricus bisporus* powder (0, 2.5, 5%), for which TPC and TFC determinations are in the range of 0.76–1.25 mg GAE/g. d. m. for the control and 5% addition of mushroom powder samples.

The antioxidant activity (DPPH and FRAP) of mushrooms and breads with mushrooms were also considered. In the available literature, DPPH and FRAP values for chaga are expressed in differentiated units; therefore, a comparison of results is problematic. Zhang et al. [37] reported that the DPPH value of chaga hydro-alcoholic extract converted to AEIO IC50 is 14.21 µg/mL. Seo and Lee [40] showed that the DPPH values range from 31.8% to 72.5%, depending on the extraction parameters. Sharpe et al. [41] showed that the % DPPH scavenged per ppm (IC50) is 0.15 for alcoholic extracts and 2.57 for aqueous extracts. Glamoclija et al. [42] showed that DPPH scavenging activity (EC50) ranges from 0.23 to 9.22 mg/mL, depending on the solvent and the country of origin of the fungus.

Based on the literature overview, no reports on the effect of the addition of chaga to bread on the DPPH and FRAP antioxidant powers were found; therefore, loaves of bread with other species of mushrooms were again taken into consideration. Sulieman et al. [36] showed an increase in the DPPH value with an increase in the content of *Agaricus bisporus* polysaccharide flour in bread. In the presented studies, the EC50 values for DPPH range from 6.85 to 19.17 mg/mL. Zhang et al. [37] showed that the DPPH value of bread with the addition of mushroom powder, expressed as free radical scavenging activity, ranges from 18.77% (control bread) to 94.49% (bread with the addition of 8%). In the work of Vlaic et al. [1], the antioxidant activity was evaluated based on DPPH free radicals, and the antioxidant activity of the fortified bread was found to have increased from 13.91% to 21.91%. Saccotelli et al. [2] showed that the DPPH value expressed as % inhibition is 4.55 (15% addition of mushroom flour in gluten-free bread). Lu et al. [38], as was mentioned previously, used dried shiitake mushrooms, sliced boletus mushrooms, and fresh champignons as an addition to white flour bread and, like other authors, showed a significant increase in antioxidant activity for bread fortified with mushrooms (increase from 0.6 to 7.2 µmol TE/g d. m.). Also, in the Sławińska et al.’s [39] paper, an increase in the antioxidant activity with the mushrooms higher amount was reported. In this study, the DPPH and FRAP antioxidant activities were in the ranges of 0.88–1.20, and 0.97–1.59 µmol/g d. m., respectively.

### 2.2. The Colour of the Bread

**Figure 1:**
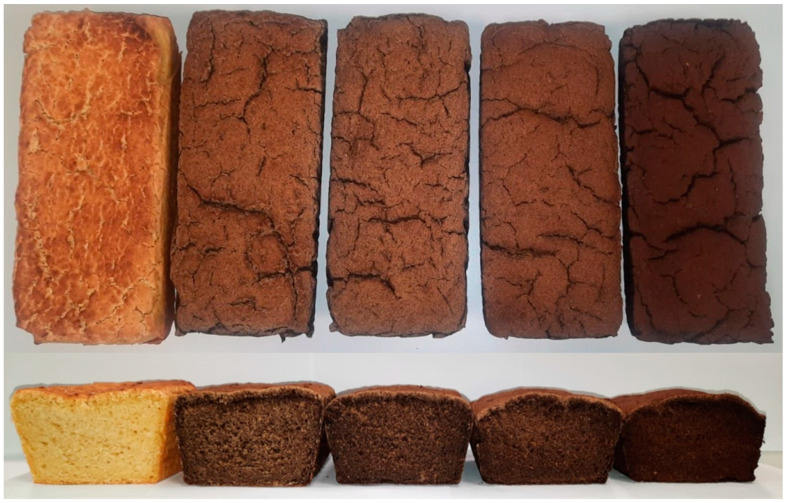
Gluten-free bread with chaga: 0, 5, 10, 15, 20% respectively (from left).

Product colour is the most important quality feature for consumer preferences. Figure 1 shows photos of the prepared baked goods, and Table 2 shows the results of the colour analysis of gluten-free bread with the addition of chaga mushroom and the control sample.

The use of the chaga mushroom additive resulted in significant changes in colour parameters (L*, a*, b*, C*). The highest lightness value (L*) measured after baking was recorded for the control bread. Bread fortified with chaga mushroom was characterized by a statistically significantly lower value of the L* parameter. The colour of the bread became darker as the level of chaga mushroom addition increased. The highest decrease in value was recorded for gluten-free bread with the addition of 20% chaga mushroom and amounted to 16.68 (there was a decrease in the value of the L* parameter by 74%), and the lowest for bread with the addition of 5%, 34.49 (there was a decrease in the value of the L* parameter by 47%). Based on the results of the ANOVA analysis and Tukey’s test (*p* > 0.05), no statistically significant differences were found for the L* parameter between gluten-free bread with 5% chaga mushroom addition and bread with 10% addition.

The lowest value of the a* parameter (indicating the intensity of the red colour) was recorded for the control bread and amounted to 1.53. The addition of chaga mushroom resulted in an increase in the value of the a* parameter. The lowest value of the a* parameter among breads fortified with chaga mushroom was recorded for bread with the addition of 15% chaga mushroom (4.55—an increase of 196% compared to the control bread), while the highest value was recorded for bread with 20% chaga mushroom addition (5.58). Based on the results of the ANOVA analysis and Tukey’s test (*p* > 0.05), no statistically significant differences were found for the a* parameter between breads containing 5%, 10%, and 15% chaga mushroom.

The highest value of the b* parameter (indicating the intensity of the yellow colour), measured after baking, was recorded for the control bread (26.79). In all cases, a decrease in the intensity of the yellow colour was noted after the addition of chaga mushroom. The highest decrease was recorded for bread with the addition of 20% chaga mushroom and amounted to 66%, and the lowest was recorded for the control bread. Based on the results of the ANOVA analysis and Tukey’s test (*p* > 0.05), no statistically significant differences were found for the b* parameter between 5% and 10% and between 15% and 20% addition of chaga flour.

In turn, the C* parameter reflecting colour saturation decreased with the increase in the addition of chaga mushroom to gluten-free bread, and the highest value of the C* parameter was recorded for the control bread. The lowest value of the C* parameter among breads fortified with chaga mushroom was recorded for bread with the addition of 20% chaga mushroom (10.59—a decrease of 61% compared to the control bread). Based on the results of the ANOVA analysis and Tukey’s test (*p* > 0.05), no statistically significant differences were found for the C* parameter between breads with 5% and 10% and 15% and 20% chaga mushroom content.

The absolute colour difference (ΔE) criterion was also used to develop the results, which indicates the differences between the colour of two samples (the higher the value, the greater the difference). In all samples of bread with the addition of chaga mushroom, the ΔE value obtained is greater than 5, which indicates a significant colour deviation (the observer gets the impression of two different colours). The highest increase in the value of the colour difference parameter after baking was recorded for bread fortified with 20% chaga mushroom addition.

Other authors [36,43] obtained similar results: bread enriched with mushroom powder or freeze-dried mushrooms was characterized by lower L* values and higher a* and b* parameters. In the study by Sulieman et al. [36], it was also shown that samples of gluten-free bread enriched with *Agaricus bisporus* mushroom polysaccharide flour and inulin had lower L* values (dark colour) of crust and crumb than the control bread, and these values were significantly lower with increasing flour and inulin content. The addition of the above-mentioned flour and inulin for gluten-free bread changed the colour of the crumb from green to red. The crust and crumb of all formulas were redder (high a* values) and yellower (high b* values) than the crust and crumb colour of the control bread. In our research, the value of the b* parameter decreased with the increase in the addition of chaga mushroom to the gluten-free bread recipe. Similar results were obtained by Zhang et al. [37], whereas the addition of *Agaricus bisporus* powder to bread increased the redness (a*) of the bread crust rose significantly, while the yellowness (b*) decreased. According to Eissa et al. [44], who conducted research on the rheological characteristics and quality analysis of bread and cookies enriched with legume or mushroom powder, even 15% of wheat flour can be replaced with sprouted legumes and mushroom powder, ensuring high protein content and good quality bread. Increasing the percentage of raw and sprouted legume and mushroom powders added to wheat flour resulted in a slight increase in the values of lightness, redness, yellowness, saturation, hue angle, and browning indices in all enriched breads and cookies.

In our study, the change in bread colour was primarily caused by the colour of the chaga flour used. As the amount of chaga flour increased, the bread became darker. Additionally, according to the observations of Ulzijargal et al. [43] and Chen et al. [45], the colour change could have been enhanced by Maillard reactions during heat treatment. This may result from a higher amount of soluble sugars and free amino acids in the mycelium compared to wheat flour. Djordjević et al. [46] explained that the crumb colour depends primarily on the colour of the ingredients used, while the crust colour is mainly the result of Maillard reactions and caramelization at high temperatures. In summary, the colour change in bread with mushroom flour was caused by the colour of the chaga flour and a combination of enzymatic browning, Maillard reactions, and caramelization.

The introduction of chaga powder into the gluten-free bread recipe resulted in a significant reduction in the whiteness index from 56.99 for sample 0 to 15.99 for sample 20. Similar trends were also observed in the case of the use of shiitake mushroom preparations. In the study by Yen et al. [47], chitin added to bread dough (5%) resulted in a product with lower brightness and whiteness index values and higher a and b values. The study by Lin et al. [48] also confirmed that replacing 5% of wheat flour in the bread recipe with shiitake flour significantly reduced the whiteness index.

The use of the chaga mushroom in gluten-free bread also resulted in a significant increase in the browning index. It is worth noting, however, that increasing the share of chaga mushrooms from 5 to 10 and 15% did not significantly affect the changes in the browning index. Generally, other studies have also shown that breads with mushroom powder added were darker and had a higher BI [39,44].

### 2.3. Textural Properties of Bread

Table 3 shows specific volume and textural properties of breads. A significant decrease in specific volume was observed after the introduction of and increase in the mushroom powder content compared to the control bread. The reason for this phenomenon was probably the ability of the dough to produce and retain gas during fermentation and baking [49]. Generally, in the studies conducted so far [43,45,50,51], in which the authors fortified wheat bread with powdered mushrooms, lower values of the specific volume were observed. However, it should be noted that the use of different raw materials affects the size of the changes. In the Sławińska et al. [39] studies, it was observed that the use of white mushrooms at an amount of 2.5% did not significantly affect the values of the specific volume, and only increasing the share of mushrooms to 5% caused a decrease in the parameter. It should also be noted that a slightly different situation was observed after using porcini mushrooms [45] as an additive to wheat bread—the introduction of 5% of this raw material resulted in a significant increase in the specific volume of the bread. However, further increasing the share of porcini mushrooms resulted in a significant decrease in this parameter.

Significant changes in the hardness, cohesiveness, and chewiness of bread were observed after the addition of chaga mushroom. Generally, the values of these parameters increased with the increasing share of the fungus.

The average hardness value increased in proportion to the increase in chaga content, up to a level of 15% addition of flour mushroom. Further increasing the amount of this raw material did not cause significant changes. Other authors [1,36,39,52] also observed that the hardness of the crumb of bread fortified with health-promoting additives; for example, boletus (*Boletus edulis*), champignon (*Agaricus bisporus*), and white and brown or lilac ear (*Auricularia auricula*) increase with the growth in the share of the tested raw material. These changes are probably related to the different chemical composition of the fungi, which may hinder the fermentation process and result in a lower specific volume and porosity of the bread and thus in its greater hardness.

A slight decrease in elasticity values was observed when chaga mushroom was introduced into the gluten-free bread recipe. However, statistical analysis did not confirm the significance of these changes. In the study by Ulziijargal et al. [43], it was observed that a small addition (5%) of dried mushrooms of different species differently affects the texture parameters of bread. The introduction of *Agaricus blazei* or *Hericium erinaceus* mushrooms into the bread recipe causes a decrease in elasticity, while after using dried *Antrodia camphorata* and *Phellinus linteus*, an increase in elasticity was observed compared to the control bread. However, in the study conducted by Lu et al. [14], the authors observed that the addition of up to 5% shiitake mushrooms and up to 10% porcini mushrooms did not cause changes in elasticity.

The cohesiveness of gluten-free bread with the addition of chaga mushroom increased compared to bread with an unmodified recipe. This increase was observed with up to 15% chaga content. In turn, increasing the share of this raw material to 20% resulted in a significant reduction in the cohesiveness of the bread crumb. Ambiguous changes in cohesiveness were also observed in the study by Sulieman et al. [36], where the addition of 3 to 9% *Agaricus bisporus* mushroom was introduced into gluten-free bread. The cohesiveness of the analysed bread decreased after the introduction of 3% fungus, then increased with 6% raw material and remained unchanged. However, the study by Ulziijargal et al. [43] showed that the addition of 5% of various mushrooms does not significantly affect changes in the cohesiveness of wheat bread.

Changes in the chewiness of gluten-free bread were analogous to changes in cohesiveness. Initially, the introduction of chaga mushroom and each subsequent increase in its share by 5% significantly increased the chewiness of the tested material. However, it was observed that the use of 20% of chaga mushroom resulted in a reduction in chewiness by 14% compared to the sample containing 15% of this raw material. Changes in chewiness, i.e., the force necessary to chew a bite of food, are closely related to changes in hardness, elasticity, and cohesiveness. Hardness is a feature whose values were higher the greater the proportion of chaga mushroom. In turn, cohesiveness and elasticity decreased after increasing the mushroom content from 15 to 20%. Similar changes were observed by Zhang et al. [37], who used the addition of two-spored mushrooms to wheat bread. The chewiness of bread increased with the increase in the addition of mushroom to 6% (14.73 N), and further increasing the share of this raw material resulted in a decrease in the chewiness value to 10.40 N.

### 2.4. Determination of Sensory Parameters of Bread

**Figure 2:**
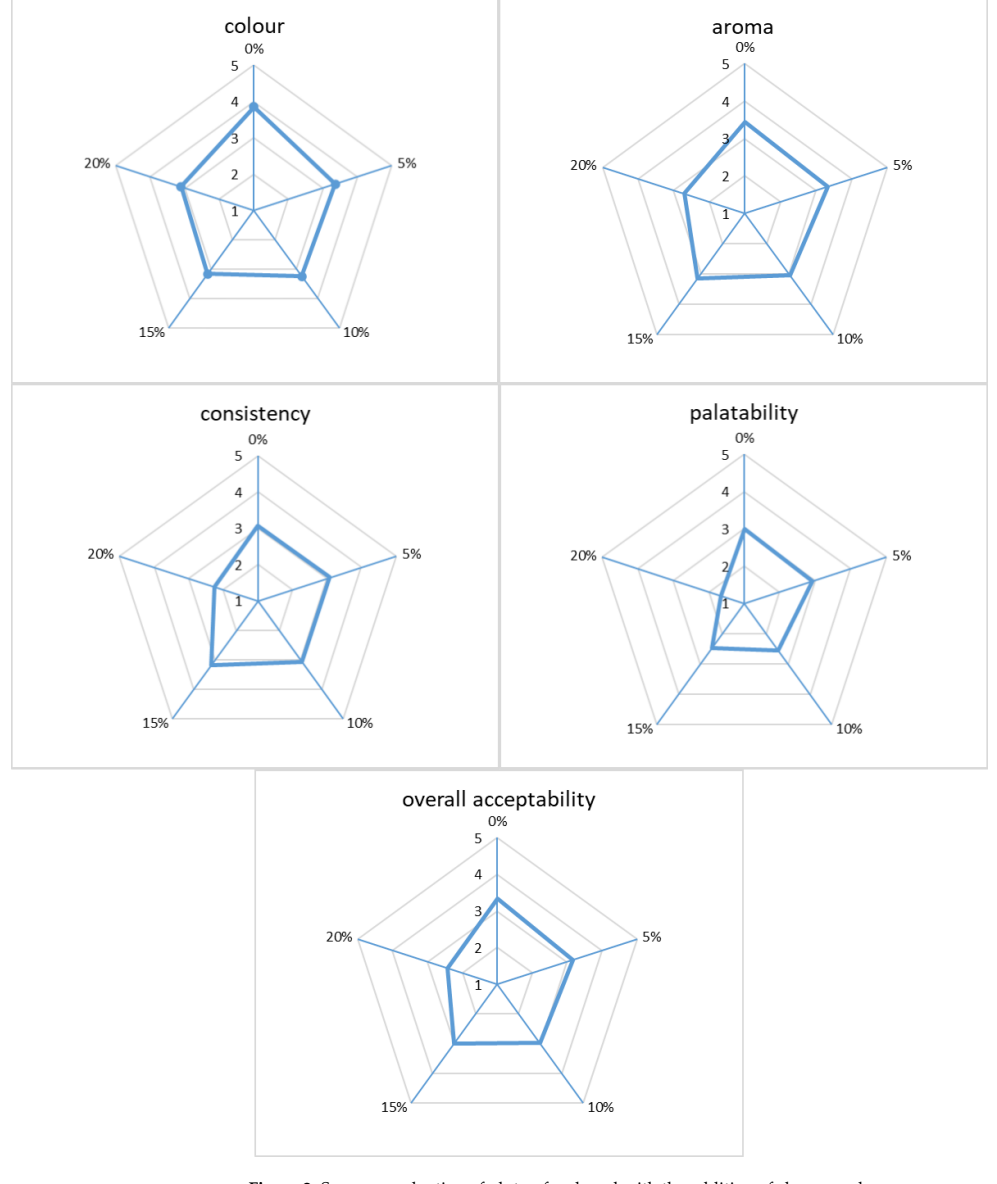
Sensory evaluation of gluten-free bread with the addition of chaga mushroom.

The results of the sensory evaluation are presented in Figure 2. Consumers rated the control gluten-free bread without the addition of chaga mushroom as the tastiest and most aromatic. The higher the addition of chaga mushroom, the lower the score for the individual parameters tested was obtained by the panellists. Consumers chose bread with 20% chaga mushroom as the least tasty (giving it a value of 1.65); this bread was also rated the worst in terms of consistency (2.25) and smell (2.71). Also, in terms of colour, the worst, according to consumers, was bread fortified with 20% chaga mushroom (3.11).

Vlaić et al. [1], by adding *Boletus edulis* mushroom flour to bread in the amounts of 3%, 6%, and 9%, showed that the bread most appreciated in terms of sensory value by consumers was the bread enriched with 6% mushroom powder. In turn, the results of the sensory analysis conducted in the work of Salehi [53] showed that mushroom powder contained in bakery products in amounts of 4–10% was characterized by the best sensory properties. In the study by Sławińska et al. [39], it was shown that the addition of up to 5% freeze-dried white or brown mushrooms to wheat bread has a positive effect on most of the analysed characteristics. Other authors have reached similar conclusions. Based on the overall acceptability score in the study conducted by Ndungu et al. [54] using bread with the addition of oyster mushroom flour, it was shown that wheat flour supplemented with 5% mushroom flour can be used to bake bread. However, all composite breads had an unattractive, dull appearance due to the presence of dark mushroom flour. Moreover, all types of fortified bread had a characteristic smell, and this could be responsible for the poor evaluation of the aroma by consumers—with the increase in the content of oyster mushrooms, the evaluation was lower. In turn, Ulziijargal et al. [43] showed that the inclusion of 5% mycelium addition to the bread recipe decreased its acceptability.

In our study, breads without mushroom flour and those with 5% chaga received the highest overall acceptability. Bread with 20% chaga received the lowest overall consumer rating, which was largely due to the palatability of the bread sample.

## 3. Materials and Methods

### 3.1. Raw Material

The dried chaga (*Inonotus obliquus*) was purchased from Dary Natury (Koryciny, Poland). The product is ecologically certificated (PL-EKO-01 Poland Agriculture certificate). The raw material was ground by a knife mill and sieved through a stainless steel sieve with a mesh size of 0.2 mm.

### 3.2. Bread Preparation Procedure

The loaves of bread with the addition of chaga were prepared based on the recipes shown in Table 4. In order to determine the amount of water necessary to prepare the dough, the water absorption (WAI) of flours and the chaga mushroom was determined: a suspension of 1 g of powdered sample was prepared in 10 mL of distilled water at room temperature for 30 min, gently stirring during this time, and then centrifuged at 3000 rpm for 15 min. The supernatant was carefully decanted, and the remaining precipitate was weighed. WAI was taken as the weight of the gel after removal of the supernatant per unit weight of the ground sample [55].

The bread dough was prepared using a laboratory spiral mixer (Ken-wood, Havant, UK), in which the ingredients were mixed for 5 min. Then, a 1075 g portion of the dough was placed in a mould and left for post-fermentation for 40 min at a temperature of 37 °C and a relative humidity of 80%. Finally, the loaves of bread were baked in a convection-steam oven (Houno, Randers, Danmark) at 230 °C for 40 min.

### 3.3. Chemical Analysis

#### 3.3.1. Extract Preparation

The extracts were prepared by adding 3 g of bread or 1 g of chaga to 30 mL of methanol and leaving them for 24 h. The next step was to pour the extract into another flask and add 30 mL of methanol to the raffinate. After next 24 h, the extracts were combined and filtered.

#### 3.3.2. Total Phenolic and Flavonoid Content Determination (TPC and TFC Assays)

The total phenolic content (TPC) in bread was determined according to the methodology described in the work of Pecyna et al. [56]. To indicate the TPC value of the mushroom, 0.8 mL of the extract was taken. The TPC7 value was calculated as mg of gallic acid equivalent per 1 g dry matter (mg GAE/g d. m.).

The total flavonoid content (TFC) value was evaluated according to the procedure presented by Kobus et al. [57]. For the determination of TFC, 2 mL of extracts was acquired and contacted with 2% AlCl_3_·6H_2_O solution (in methanol) The TFC value was calculated as mg of quercetin equivalent per 1 g dry matter (mg QE/g d. m.).

#### 3.3.3. Antioxidant Activity Analysis (DPPH and FRAP Assays)

The antioxidant activity studies were performed using DPPH and FRAP reagents. The mushroom activity expressed by 2,2-diphenyl-1-picrylhydrazyl (DPPH) was determined according to the methodology described in the work by Pecyna et al. [56]. To determine the DPPH activity of bread, 420 µL of the extract was taken.

The ferric reducing antioxidant power (FRAP) activity determination was per-formed according to the modified procedure of Benzie and Strain [58] described by Kobus et al. [59]. For this purpose, the solutions of 300 µL of bread extracts with 5 mL of FRAP reagent were prepared. For chaga extract, the solution was diluted (*v*/*v* = 1:1). In both cases, the antioxidant activities were calculated as µg Trolox per g of dry matter (µg/g d. m.).

#### 3.3.4. The Physical Properties of Bread

Six hours after baking, the physical parameters of the bread were examined. Specific volume (cm^3^ g^−1^) was calculated as the ratio of bread volume to bread mass. The rapeseed displacement method was used to determine the bread volume. All these assessments were repeated three times [60].

### 3.4. Determination of Textural Properties of Bread

Six hours after baking, the bread crumb texture was analysed (TPA test). A testing machine (Zwick/Roel, Z0.5, AG, Ulm, Germany) with test Xpert II software was used for the tests. During measurement, the product was compressed twice to 50% of its original height, with a 50 kg load cell. The speed of the compression punch was 50 mm·min^−1^. Compression was performed using a flat cylindrical punch with a diameter of 100 mm. In order to prepare the samples for testing, a sample measuring 20 × 20 × 10 mm was cut from the middle part of a 10 mm thick slice. The test determined the following values: hardness [N], elasticity [-], cohesiveness [-], and chewiness [N].

### 3.5. Colour Analysis

The colour was determined using a 3Color^®^ SF80 spectrophotometer (Marcq-en-Barœul, France). Each sample was analysed in 10 repetitions (light source: D65, observer: 10°, measuring head opening: 8 mm). The results were expressed in the CIELab system, and the following parameters were measured: L*—brightness, a*—colour change from green to red, and b*—colour change from blue to yellow (higher values of a* and b* mean greater intensity of red and yellow, respectively). Additionally, the total colour change (absolute colour difference) ΔE between gluten-free bread (control) and bread with the addition of chaga mushroom was calculated using the following formula:

(1) $$\Delta E=\sqrt{{\left(\Delta L\right)}^{2}+{{\left(\Delta a\right)}^{2}+(\Delta b)}^{2}}$$

where ΔL, Δa, and Δb are indices of the difference in the colour of the surfaces of samples compared with the control bread [44].

The whiteness index (WI) and browning index (BI) of gluten-free bread were calculated based on the measured parameters (L*, a* and b*) according to Equations (2) and (3), respectively [47].

(2) $$WI=100-\sqrt{{\left(100-L*\right)}^{2}+{a*}^{2}+{b*}^{2}}$$

(3) $$BI=\frac{100\cdot (x-0.31)}{0.172}$$

where

(4) $$x=\frac{a*+1.75\cdot L*}{5.645\cdot L*+a*-3.012\cdot b*}$$

### 3.6. Determination of Sensory Parameters of Bread

Breads with the addition of chaga mushroom were rated by a panel of 55 trained consumers selected among employees and students (aged 20–64) of the University of Life Sciences in Lublin. The selection criteria were good health, non-smokers, and voluntary participation. The test was performed in the laboratory under LED lighting and at ambient temperature. The panellists were provided with mineral water as a neutralizing agent. The samples were administered in random order. The assessment was carried out immediately after the baking and cooling of the breads. The panellists assessed the colour, smell, consistency (elasticity and porosity) and palatability of the bread crumb. The results were presented on a 5-point structural scale (1—“I don’t like it very much” and 5—“I like it very much”).

### 3.7. Statistical Analysis

The obtained results were subjected to analysis of variance (ANOVA). The significance of the variations among the mean values was assessed using Tukey’s test at a significance level of *p* < 0.05. All statistical data treatments were performed with Statistica software (Statistica 13; StatSoft Inc., Tulsa, OK, USA).

## 4. Conclusions

Gluten-free bread with the addition of chaga flour was characterized by a significant increase in the content of bioactive substances and antioxidant activity compared to the control bread. The introduction of chaga added increased the content of polyphenols (78%) and flavonoids (81%), as well as the ability to scavenge free radicals (DPPH—238%) and the antioxidant capacity, expressed as the ability to reduce iron (III) ions (FRAP—199%).

The addition of chaga flour also significantly influenced changes in bread colour parameters. Increasing the amount of chaga resulted in a gradual darkening of the bread (decrease in L* value). At the same time, the values of parameters a* (intensity of red colour) and b* (intensity of yellow colour) increased. Textural analysis showed that the hardness, consistency, and chewiness of the bread increased with the increasing content of mushroom flour. No statistically significant changes in bread elasticity were found. In the sensory evaluation, the control bread received the highest marks for taste and aroma. Increasing the addition of chaga flour resulted in a gradual decrease in scores for taste, texture, and smell. Bread with 20% chaga flour added was rated the lowest in all categories, receiving the lowest overall acceptability.

The addition of chaga flour to gluten-free bread significantly increased the content of bioactive compounds and antioxidant properties, which may have a positive impact on consumers’ health. However, high levels of chaga flour (15–20%) negatively impacted the colour, texture, and sensory characteristics of bread, suggesting an optimal chaga flour addition level of approximately 5–10% to balance health benefits and consumer acceptance.

## Figures and Tables

**Table 1:** Chemical analysis of chaga-doped breads.

Probe	TPC [mg GAE/ g d. m.]	TFC [mg QE/ g d. m.]	DPPH [μg TE/ g d. m.]	FRAP [μg TE/ g d. m.]
0	0.861 ± 0.025 ^a^	0.070 ± 0.001 ^a^	0.670 ± 0.070 ^a^	1.124 ± 0.004 ^a^
5	0.936 ± 0.020 ^a^	0.098 ± 0.001 ^b^	1.049 ± 0.026 ^b^	1.718 ± 0.017 ^b^
10	1.065 ± 0.052 ^b^	0.103 ± 0.001 ^c^	1.375 ± 0.022 ^c^	2.095 ± 0.032 ^c^
15	1.258 ± 0.033 ^c^	0.119 ± 0.001 ^d^	1.717 ± 0.037 ^d^	2.530 ± 0.042 ^d^
20	1.532 ± 0.066 ^d^	0.127 ± 0.001 ^e^	2.263 ± 0.055 ^e^	3.360 ± 0.023 ^e^

0—control probe, 5—gluten-free bread with the addition of 5% chaga; 10—gluten-free bread with the addition of 10% chaga; 15—gluten-free bread with the addition of 15% chaga; 20—gluten-free bread with the addition of 20% chaga. Data values of each parameter with different superscript letters in rows are significantly different (Tukey test, *p* ≤ 0.05).

**Table 2:** Colour parameters obtained from the crumb of gluten-free bread with the addition of chaga mushroom.

Probe	L*—Value	a*—Value	b*—Value	C*—Value	ΔE—Value	WI	BI
0	64.49 ± 1.46 ^a^	1.53 ± 0.40 ^a^	26.79 ± 0.95 ^a^	26.84 ± 0.96 ^a^		56.99 ± 1.47 ^a^	51.37 ± 3.48 ^a^
5	34.49 ± 2.74 ^b^	4.97 ± 0.16 ^b^	15.00 ± 0.76 ^b^	15.80 ± 0.77 ^b^	33.92	32.60 ± 2.52 ^b^	65.92 ± 4.59 ^b^
10	32.76 ± 0.97 ^b^	4.99 ± 0.22 ^b^	15.47 ± 0.56 ^b^	16.26 ± 0.59 ^b^	35.30	30.82 ± 0.94 ^b^	72.70 ± 4.25 ^b^
15	23.41 ± 1.47 ^c^	4.55 ± 0.37 ^b^	10.06 ± 1.53 ^c^	11.05 ± 1.53 ^c^	45.70	22.60 ± 1.28 ^c^	68.38 ± 7.68 ^b^
20	16.68 ± 1.99 ^d^	5.58 ± 0.73 ^c^	9.00 ± 1.65 ^c^	10.60 ± 1.77 ^c^	51.98	15.99 ± 1.87 ^d^	99.64 ± 19.69 ^c^

0—control probe, 5—gluten-free bread with the addition of 5% chaga mushroom; 10—gluten-free bread with the addition of 10% chaga mushroom; 15—gluten-free bread with the addition of 15% chaga mushroom; 20—gluten-free bread with the addition of 20% chaga mushroom; WI—whiteness index; BI—browning index. Data values of each parameter with different superscript letters in rows are significantly different (Tukey test. *p* ≤ 0.05).

**Table 3:** Specific volume and textural properties of breads.

Probe	Specific Volume [cm^3^·g^−1^]	Hardness [N]	Elasticity [-]	Cohesiveness [-]	Chewiness [N]
0	1.223 ± 0.003 ^a^	17.54 ± 1.40 ^a^	0.754 ± 0.071 ^a^	0.132 ± 0.011 ^a^	1.74 ± 0.20 ^a^
5	1.165 ± 0.004 ^b^	23.88 ± 1.63 ^b^	0.734 ± 0.025 ^a^	0.180 ± 0.016 ^b^	3.13 ± 0.35 ^b^
10	1.117 ± 0.010 ^c^	32.26 ± 3.17 ^c^	0.707 ± 0.035 ^a^	0.194 ± 0.011 b ^c^	4.40 ± 0.35 ^c^
15	1.053 ± 0.012 ^d^	49.32 ± 1.62 ^d^	0.706 ± 0.043 ^a^	0.228 ± 0.016 ^c^	7.93 ± 0.69 ^e^
20	0.966 ± 0.019 ^e^	52.44 ± 3.82 ^d^	0.673 ± 0.061 ^a^	0.190 ± 0.031 ^b^	6.88 ± 0.78 ^d^

0—control probe, 5—gluten-free bread with the addition of 5% chaga mushroom; 10—gluten-free bread with the addition of 10% chaga mushroom; 15—gluten-free bread with the addition of 15% chaga mushroom; 20—gluten-free bread with the addition of 20% chaga mushroom. Data value of each parameter with a different superscript letter in rows are significantly different (Tukey test. *p* ≤ 0.05).

**Table 4:** The composition of bread dough with chaga addition.

Probe Code	Bread Composition (g)								
	Rice Flour	Corn Flour	Potato Starch	Rape Seed Oil	Dry Yeast	Salt	Sugar	Ground Flax Seeds	Chaga
0	250	200		30	8	12	10		0
5	225								25
10	200		50					15	50
15	175								75
20	150								100

## Data Availability

Data are contained within the article.
